# LIM homeodomain transcription factor *Isl1* affects urethral epithelium differentiation and apoptosis via *Shh*

**DOI:** 10.1038/s41419-019-1952-z

**Published:** 2019-09-26

**Authors:** Tiantian Su, Hui Liu, Di Zhang, Guojin Xu, Jiali Liu, Sylvia M. Evans, Jirong Pan, Sheng Cui

**Affiliations:** 1 State Key Laboratory of Agrobiotechnology, College of Biological Sciences, China Agricultural University, 100193 Beijing, People’s Republic of China; 2 College of Veterinary Medicine, Yangzhou University, 225009 Yangzhou, Jiangsu People’s Republic of China; 3 Skaggs School of Pharmacy, University of California San Diego, 9500 Gilman Drive, La Jolla, CA 92093 USA; 4 Key Laboratory of Human Disease Comparative MedicineInstitute of Laboratory Animal Science, Chinese Academy of Medical Science and Comparative Medical Center, Peking Union Medical College, 100021 Beijing, People’s Republic of China

**Keywords:** Apoptosis, Physiology

## Abstract

Urethral hypoplasia, including failure of urethral tube closure, is one of the common phenotypes observed in hereditary human disorders, the mechanism of which remains unclear. The present study was thus designed to study the expression, functions, and related mechanisms of the LIM homeobox transcription factor *Isl1* throughout mouse urethral development. Results showed that *Isl1* was highly expressed in urethral epithelial cells and mesenchymal cells of the genital tubercle (GT). Functional studies were carried out by utilizing the tamoxifen-inducible *Isl1*-knockout mouse model. Histological and morphological results indicated that *Isl1* deletion caused urethral hypoplasia and inhibited maturation of the complex urethral epithelium. In addition, we show that *Isl1*-deleted mice failed to maintain the progenitor cell population required for renewal of urethral epithelium during tubular morphogenesis and exhibited significantly increased cell death within the urethra. Dual-Luciferase reporter assays and yeast one-hybrid assays showed that ISL1 was essential for normal urethral development by directly targeting the *Shh* gene. Collectively, results presented here demonstrated that Isl1 plays a crucial role in mouse urethral development, thus increasing our potential for understanding the mechanistic basis of hereditary urethral hypoplasia.

## Introduction

External genital development of mammals is regulated by a sophisticated progression of budding and fusion events. In mice, paired genital swellings arise lateral to the cloacal membrane at around embryonic day (E) 10.5, then these swellings merge to form a single genital tubercle (GT) at E11.5, and finally give rise to the prepuce by E13.5^1^. Up to E15.5, male and female GTs are morphologically indistinguishable, and their development is presumably controlled by the same genetic program called androgen-independent GT patterning^2–4^. After E16.5, urethra in males canalize in the presence of androgen signaling, whereas they remain as epithelial cords in females^5,6^. Three germ layers participate in external genital development: endodermally derived urethral plate epithelium forms the entire urethral tube, mesoderm forms the stromal tissue of the genital, and a jacket of ectodermal epithelium forms the skin^7–9^. Epithelial differentiation is an important process for reproductive organ development. Urethral defects lead to severe developmental disease; a prime example being the most common urethra defect in human, hypospadias, a failure of urethral tube closure often accompanied by agenesis of the ventral aspect of the genitals, which affects ∼1 in 250 live births^10,11^.

Genetic mutation is one of the crucial causes of urethral defects. Up to now, several factors have been identified that participate in urethral development. Sonic hedgehog (*Shh*) mutants result in hypospadias associated with increased cell death and failure to maintain Wnt-Fgf8 signaling in endoderm^2,7,12–15^. The *Fgf8*-expressing distal urethral epithelium has been shown to have a growth-promoting function^16,17^. *Fgfr2* is required for outgrowth and patterning of the GT, and regulates cell number by controlling the length of specific cell cycle phases^10,18,19^. *Hoxa13* mutant hypospadias occur as a result of combined loss of *Fgf8* and *Bmp7* expression in the urethral epithelium^16^. Genes including *Lef1*^20,21^, *Msx1*^1^, *b-catenin*^22^, and *Noggin*^6^ also have been shown to be involved in urethral development. However, a comprehensive network of gene regulation during urethral development remains unclear.

The LIM homeobox gene *Isl1* plays critical roles in multiple tissues in mouse embryonic development, including the nerve^23,24^, stomach^25,26^, limb^27,28^, and heart^29–32^, by functioning in cell proliferation, apoptosis^33^, and differentiation^34,35^. ISL1 is also involved in hormone biosynthesis and secretion in endocrine tissues such as pancreatic islets^36,37^, pituitary^38^, and pineal glands^39,40^. *Isl1* expression has been detected in genital mesenchyme, and *Isl1* knockout in mesenchyme results in urogenital malformations, although urethral development in these *Isl1* mutants was unaffected^41^. Additionally, genome-wide association studies have revealed that *Isl1* is a major susceptibility gene for human congenital anomalies of the kidney and urinary tract (CAKUT) and bladder exstrophy-epispadias complex (BEEC)^42–44^, implying potential roles for *Isl1* in urethra development. The present study was thus designed to examine a potential role for the transcription factor *Isl1* in urethral development.

## Materials and methods

### Mice maintenance and treatment

Adult (6- to 8-week-old) male and female C57BL/6 mice were used for this study. The age of embryos was determined by the appearance of the vaginal plug, which was taken to be E0.5.

Generations of *Isl1*^*MCM/Del*^ and *Isl1*^*F/+*^ mice were described previously^45^. Briefly, we used a “floxed” *Isl1* allele (*Isl1F*) in which LoxP sites were inserted into the introns flanking exon 4 of the *Isl1* locus, and a tamoxifen-inducible knock in *Isl1-MERCreMER* allele. *Isl1*^F/F^ mice were mated with *Isl1*^MCM/+^ mice to generate litters with equal numbers of *Isl1*^MCM/F^-inducible knockouts and *Isl1*^*F/+*^ controls. To induce excision in *Isl1*^MCM/F^ embryos, pregnant females were administered an oral gavage of 75 mg/kg body weight of tamoxifen (T5648; Sigma, St. Louis, MO, USA) in corn oil (10 mg/ml) at E9.5 for three consecutive days just before *Isl1* expression sharply increased. Embryos were harvested from pregnant mice obtained by timed matings at the desired stages of development and genotyped by common PCR. Sex of embryos were identified by common PCR before E15.5^46^ and later morphological analysis of gonads. For studies involving embryos, only males were presented (except for specially marked parts). All animal studies were approved by Ethics Committee of China Agricultural University and performed in accordance with the guidelines and regulatory standards of the Institutional Animal Care and Use of Animals for Scientific Purposes.

### Common PCR and qPCR

GTs were dissected from stage-matched embryos and were pooled according to genotype for each litter collected. Total RNA was extracted using Trizol reagent (9109; TaKaRa, Dalian, China). RNA quantity and purity were determined using a NanoDrop (ND-2000, USA). One microgram of high-quality RNA (260/280 ratios slightly higher than 2.0 and 260/230 ratios higher than 1.7 for each pooled sample was reverse transcribed into complementary DNA (cDNA) using M-MLV (M170A; Promega, USA). Quantitative PCR (qPCR) amplication was performed four times (DRR420A; Takara, Dalian, China) in the ABI 7500 system (Applied Biosystems, Foster City, USA). For normalization purposes, an identical set of reactions were prepared for glyceraldehyde 3-phosphate dehydrogenase (GAPDH).

Genomic DNA was isolated from tail or GT following the HotSHOT method^47^ and genotyping was performed using standard PCR methods with specific primers^45^. The relative location of primers used to identify the wild-type (WT), floxed (Flox), and rearranged alleles are shown as solid arrows in Fig. 2b; primer pair F1/R1 amplifies a 406 bp WT and 502 bp Flox bands. In *Isl1*^*MCM/Del*^ mice, the expected PCR products using the primer pairs *MCM-F* and *MCM-R* were 289 bp. Primer pair F1/R2 amplifies a 730-bp rearranged floxed allele in *Isl1*^*MCM/Del*^ genital after Cre activation; there was no product under the same PCR condition before Cre activation. The common PCR (Bio-Rad Laboratories) was performed using the following protocol: 95 °C for 5 min; 95 °C for 30 s, 60 °C for 30 s, 72 °C for 45 s (35 cycles); 72 °C for 5 min; 4 °C holding. PCR products were observed on a 1.5% agarose gel. PCR primers designed for this study were listed in Supplementary Tables S1 and S2.

### Western blot

Briefly, total proteins were extracted in RIPA (radio immunoprecipitation assay) buffer (9806; Cell Signaling, Danvers, MA, USA) containing 1 mM phenylmethylsulfonyl fluoride (8553S; Cell Signaling, Danvers, MA, USA) according to the manufacturer’s protocol. The BCA Protein Assay Kit (HX18651; Hoaxing, China) was used to measure protein concentration. Electrophoresis was performed with 30 μg total proteins separated by 12% sodium dodecyl sulfate-polyacrylamide gel electrophoresis and transferred to polyvinylidene difluoride membranes (IPVH00010, Millipore, USA). The membrane was blocked with 5% (w/v) nonfat dry milk in 0.05 M Tris-buffered saline and 0.1% Tween-20 (TBST, pH 7.4) for 1 h and incubated with anti-ISL1(1:300; AF1837; R&D, Minneapolis, MN, USA) antibody and internal control GAPDH antibody (1:10,000; Ambion, USA) overnight at 4 °C. The secondary antibody, horseradish peroxidase-conjugated donkey anti-goat IgG (1:10,000; ab205723; Abcam, Cambridge, MA, USA), was diluted 1:5000 in TBST. The membranes were visualized using the SuperSignal West Pico Kit (Thermo Scientific, Waltham, MA, USA) substrate at room temperature. We used the ImageJ Software to assay the relative intensity of each blot. The intensity values of each group were normalized to GAPDH (internal control) in the same group.

### Hematoxylin and eosin staining

The embryonic specimens were fixed overnight in 4% paraformaldehyde/phosphate-buffered saline (PBS), dehydrated in ethanol, and embedded in paraffin. Sections of 5 μm were cut from wax-embedded embryos and floated onto slides coated with 3-triethoxysilylpropylamine (440140, Sigma). The slides were dried at 37 °C overnight, dewaxed through xylene, and then rehydrated through decreasing concentrations of ethanol. Next, they were stained with hematoxylin, counterstained with eosin, dehydrated, and equilibrated with xylene. Sections were photographed under bright-field microscope photograph system (Leica Microsystems, Buffalo Grove, IL, USA).

### Immunofluorescence and immunohistochemistry

The sections were deparaffinized, rehydrated, and subjected to microwave antigen retrieval with 0.01 M sodium citrate buffer (pH 6.0). The sections were then blocked with 10% normal donkey or goat serum in PBS at room temperature for 1 h, followed by the incubation with anti-ISL1 antibody (1:50; 40.2D6; Developmental Studies Hybridoma Bank, Iowa City, IA, USA), anti-bromodeoxyuridine (BrdU) (1:300; G3G4; Developmental Studies Hybridoma Bank) overnight at 4 °C. After rinsing thoroughly with PBS, the sections were incubated with the secondary antibodies, which were cy3-conjuncted goat anti-mouse IgG (1:50; 115-165-003; Jackson ImmunoResearch, West Grove, PA, USA) for 2 h at room temperature. The sections were then rinsed with PBS and stained with 4′,6-diamidino-2-phenylindole (10236276001; Roche Applied Science, Basel, Switzerland) for 10 min. Finally, 20 μl Vectashield mounting medium (H-1000; Vector Laboratories, Burlingame, CA, USA) was applied to each slide, and a coverslip was sealed in place. Leica Microsystems was used for imaging immunofluorescent sections. IgG was used as the negative control.

For immunohistochemistry (IHC), the general procedure was similar to immunofluorescence, the endogenous peroxidase was inactivated with 3% hydrogen peroxide before antigen retrieval. Anti-ISL1 antibody was applied overnight at 4 °C. A biotinylated goat anti-mouse secondary antibody (1:200; 115-065-146; Jackson ImmunoResearch, West Grove, PA, USA) was applied for 2 h at room temperature followed by streptavidin peroxidase (1:200; 123-065-021; Jackson ImmunoResearch, West Grove, PA, USA) for 2 h. Diaminobenzidine (D4293; Sigma, St. Louis, MO, USA) and 0.1% H_2_O_2_ were used for detecting peroxidase activity.

### Detection of proliferation and apoptotic cells

To detect the proliferation of urethral cells, pregnant mice were injected intraperitoneally with BrdU (100 mg/kg body weight) 4 h before sacrifice. Embryos were removed and processed for immunofluorescence staining, following the procedure described above. Terminal deoxynucleotidyl transferase dUTP nick-end labeling (TUNEL) assay for the detection of apoptotic cells was performed with the In Situ Apoptosis Detection Kit (Roche Applied Science) according to the manufacturer’s instructions. Four embryos of each group were included for the analysis, and six sections of each embryo were examined for the proliferation and apoptosis.

### Transient transfection and Dual-Luciferase reporter assays

The *Shh*, *Fgfr2*, and *Fgf8* promoter fragment (0 to −2000 bp) was cloned from mouse genomic DNA and inserted into the pGL3.0 vector (E1751, Promega). *Isl1* was amplified using the primers containing the *Nhe*I–*Xho*I restriction sites and inserted into the pcDNA3.1 vector. An empty luciferase reporter vector was used as a control. The primer sequence is in Supplementary Table S3.

The human embryonic kidney 293FT cells were maintained in Dulbecco’s modified Eagle’s medium supplemented with 10% fetal bovine serum and 1% penicillin–streptomycin at 37 °C with 5% CO_2_. Cells were transfected with *Isl1* expression vector, *Fgf8*, *Fgfr2*, *Shh* luciferase reporter vector, and pTK-Ranilla vector using Lipofectamine 2000 (Invitrogen) according to the manufacturer’s instruction. Twenty four hours post transfection, luciferase activity was measured by using Dual-Luciferase Reporter Assay Kit (E1910; Promega) on a Modulus II Microplate Multimode Reader (Turner Biosystems, Sunnyvale, CA, USA). The values were normalized against *Renilla* luciferase activity. At least four independent experiments were performed.

### Yeast one-hybrid assay

The *Fgf8*, *Fgfr2*, *Shh* promoter fragment (0 to −2000 bp) was inserted into the pLacZi vector and *Isl1* fragments was inserted into the pB42AD vector as described previously. The primer sequence is in Supplementary Table S4. Plasmids for pB42AD DNA-binding domain fusions (BD) were co-transformed with the pLacZi:LacZ reporter gene plasmid (AD) into the yeast strain EGY48 using standard transformation techniques. Transformants were grown on proper dropout plates containing X-gal (5-bromo-4-chloro-3-indolyl-β-d-galactopyranoside) for color development. Yeast transformation and liquid assay were conducted as described in the Yeast Protocols Handbook (Clontech). At least four independent experiments were performed.

### Statistics

The results were expressed as means ± SEM of at least four independent experiments. The differences among groups were determined using a Student’s *t* test or a one-way analysis of variance. *P* < 0.05 was considered to be statistically significant.

## Results

### *Isl1* expression in the developing mouse urethra

**Fig. 1:**
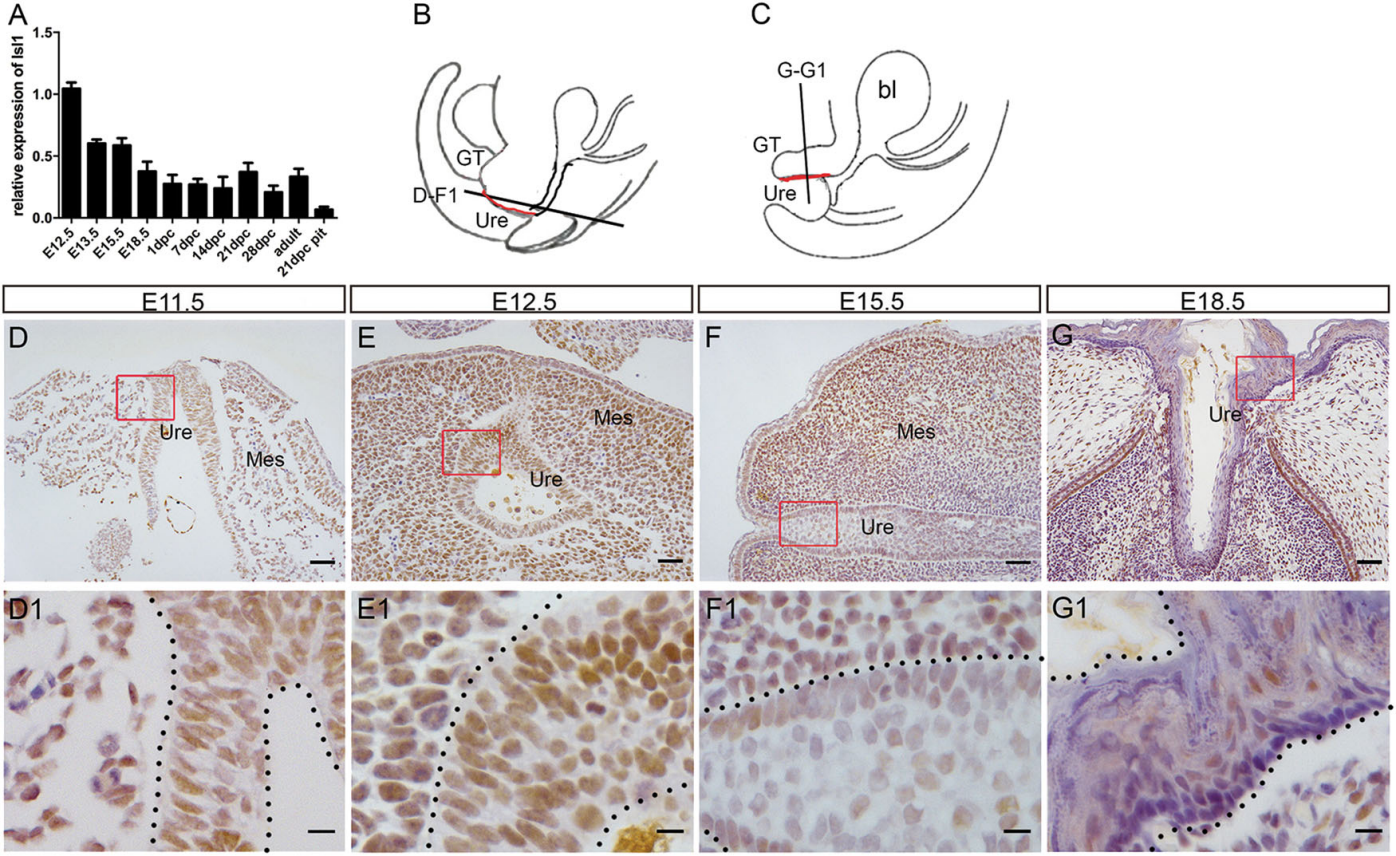
*Isl1* expression pattern during GT urethral development. **a** *Isl1* mRNA relative expression levels at different developmental stages in male GTs. Twenty-one-day mice pituitaries served as a positive control. Each sample contained at least four independent replications. Data were presented as means ± SEM. **b**, **c** Abridged general view of **d**–**g1** slice position. Red line represents the urethra. **d**–**g**1 ISL1 localization in GTs at E11.5, E12.5, E15.5, and E18.5. Scale bars: **d**–**g**, 50, μm; **d1**–**g1**, 10 μm. GT, genital tubercle; Mes, genital mesenchyme; Ure, urethra; bl, bladder

First, we examined *Isl1* messenger RNA (mRNA) levels in developing male mouse urethra from E12.5 to adult by using qPCR. Results showed that *Isl1* mRNA levels were highest at E12.5, the earliest stage examined, decreasing by 40% at E13.5 and E15.5, and by 62.5% at E18.5 relative to levels observed at E12.5, followed by no significant changes up until adult stages (Fig. 1a). In addition, we investigated ISL1 expression by IHC, and results showed that ISL1 was weakly stained in the outgrowth at E11.5 (Fig. 1d, d1). At E12.5 and E15.5, ISL1 was strongly stained in most urethral epithelium (Fig. 1e, f, e1, f1) and around mesenchymal cells, but by E18.5, only a few ISL1-positive cells were observed within the urethral epithelium (Fig. 1g, g1). This *Isl1* expression pattern in during urethral development suggests that *Isl1* might be involved in regulating urethral development.

### *Isl1* expression is effectively ablated in *Isl1*^***MCM/Del***^ mice

**Fig. 2:**
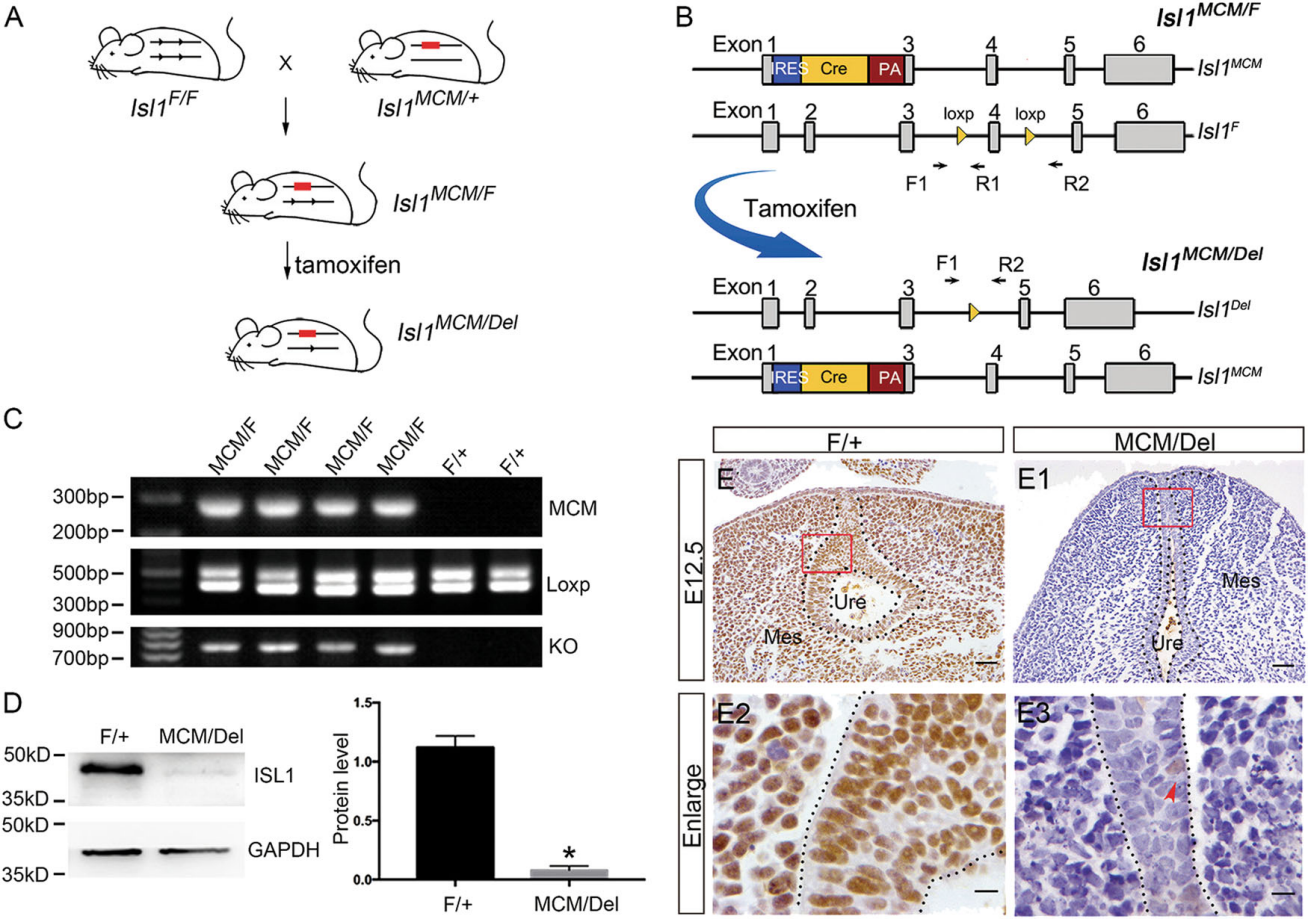
*Isl1* knocked down efficiency at E12.5 urethra in *Isl1*-inducible knockout embryos. **a** Schematic of inducible knockout mouse. **b** *Isl1* gene knockout strategy and location of primers used for PCR verification of knockout genotype. **c** Genotype of *Isl1*^*MCM/F*^ mutants and *Isl1*^*F/+*^ controls assayed by common PCR. **d** Representative image of Western blot detecting the knockdown efficiency of ISL1 protein. **e–e3** ISL1 expression in urethral epithelium of *Isl1*^*MCM/Del*^ and control embryos. Scale bars: **e**–**e1**, 50 μm; **e2**–**e3**, 10 μm. Red arrowheads representative ISL1-positive cells. Results were presented as means ± SEM. Each test contained at least four independent replications for Western blot and PCR. *P < 0.05

To identify whether *Isl1* was involved in regulating urethral development, the tamoxifen-inducible Cre/loxP system was utilized to knockout *Isl1* in mouse as in our previous report^25^. Briefly, *Isl1-MERCreMER* males^45^ were used to mate with *Isl1*^*loxP/loxP*^ female mice^48^ to produce embryos carrying *Isl1 mER-Cre-mER*; *Isl1* loxP/+ (Fig. 2a), where the *Isl1* promoter drives the expression of Cre. We then injected tamoxifen into pregnant dams at E9.5 to generate *Isl1*-inducible knockout embryos, hereafter referred to as *Isl1*^*MCM/Del*^. *Isl1*^*F/+*^ sibling embryos served as controls. Genotype of control and mutant embryos were detected by PCR (Fig. 2c). Western blot results showed that ISL1 protein levels in knockout mice decreased more than 90% compared with control embryos (Fig. 2d). IHC results demonstrated that ISL1 staining was reduced in urethral epithelium of *Isl1*^*MCM/Del*^ mice compared to controls (Fig. 2e–e3). These data demonstrated that *Isl1* was effectively down-regulated in urethral epithelium of *Isl1*^*MCM/Del*^ embryos.

### *Isl1* deletion disrupts urethral internalization

**Fig. 3:**
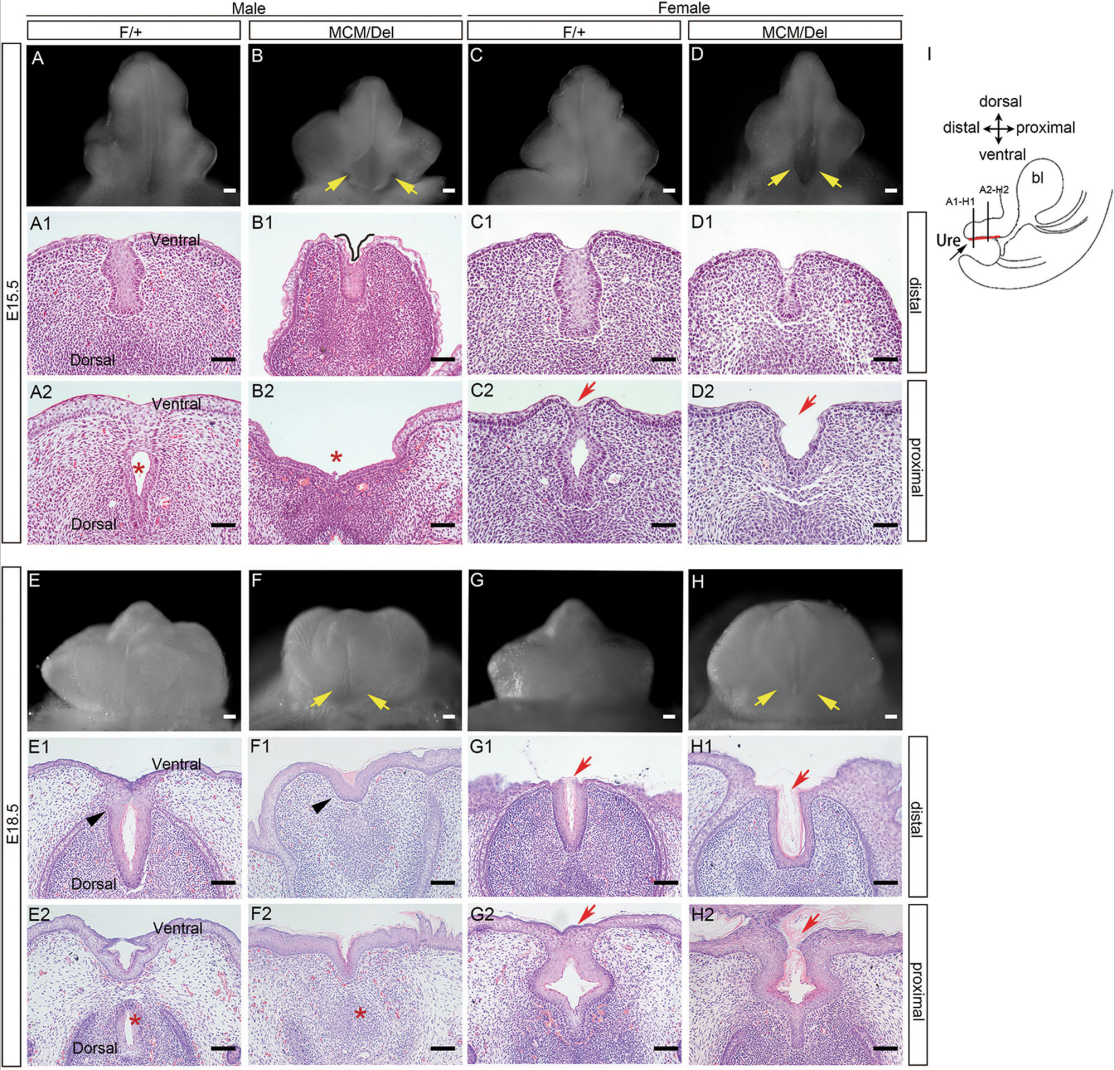
Abnormalities of *Isl1*^*MCM/Del*^ embryos in urethral development. **a**–**h** Morphology changes of mutant and control GTs. Yellow arrow indicates abnormal urethral opening. **a1**–**h2** HE-stained cross-sections for the distal and proximal regions of the GTs. The internal tubular urethra was absence pointed by red asterisks and black asterisks in male. Urethral tube openings were pointed by red arrows in female. **i** Abridged general view of **a1**–**h2** slice position. Red line represents the urethra. Scale bar: **a**–**h**, 100 μm; **a1**–**h2**, 50 μm

To investigate effects of *Isl1* on GT urethral development, morphological and histological examinations were performed at E15.5 and E18.5 for *Isl1*^*MCM/Del*^ embryos and control littermates. *Isl1* deletion resulted in hypoplasia of the GT in male and female embryos (Fig. 3), while GTs had developed with a urethral seam along the ventral midline in control male embryos at E15.5 (Fig. 3a). The overall size of mutant GTs was smaller than control GTs. In addition, the proximal urethral opening of mutants was both wider mediolaterally and extended farther proximodistally compared with controls (Fig. 3b). By E18.5, in *Isl1*^*MCM/Del*^ male embryos, the fusion of the GT prepuce failed, which led to an ectopic opening (Fig. 3e, f).

Further histological analyses revealed that at E15.5, the proximal urethral plate of control embryos had separated medially to form a urethral tube (Fig. 3a2), whereas the distal end remained a bilaminar epithelial plate (Fig. 3a1). In *Isl1*^*MCM/Del*^ mutants at the same stage, urethral tube epithelium was not detected proximally (Fig. 3b2), but the urethral remnant and a shallow groove on the ventral surface of the genital distal were observed (Fig. 3b1). By E18.5, *Isl1* deletion resulted in the absence of internal urethra of male GTs (Fig. 3, compare e1 with f1, Fig. 3, compare e2 with f2).

Female *Isl1*^*MCM/Del*^ mutants had hypoplasia similar to that observed in males, but effects on prepuce fusion were not as severe as those observed in male mutants either at E15.5 (Fig. 3c, d) or E18.5 (Fig. 3g, h). *Isl1* deletion resulted in an open ventral urethra in females (Fig. 3, compare c2 with d2 at E15.5 and compare g2 with h2 at E18.5). Collectively, these data demonstrated that *Isl1* was crucial for urethral formation of GTs.

### *Isl1* affects epithelial stratification and urethral cell differentiation

**Fig. 4:**
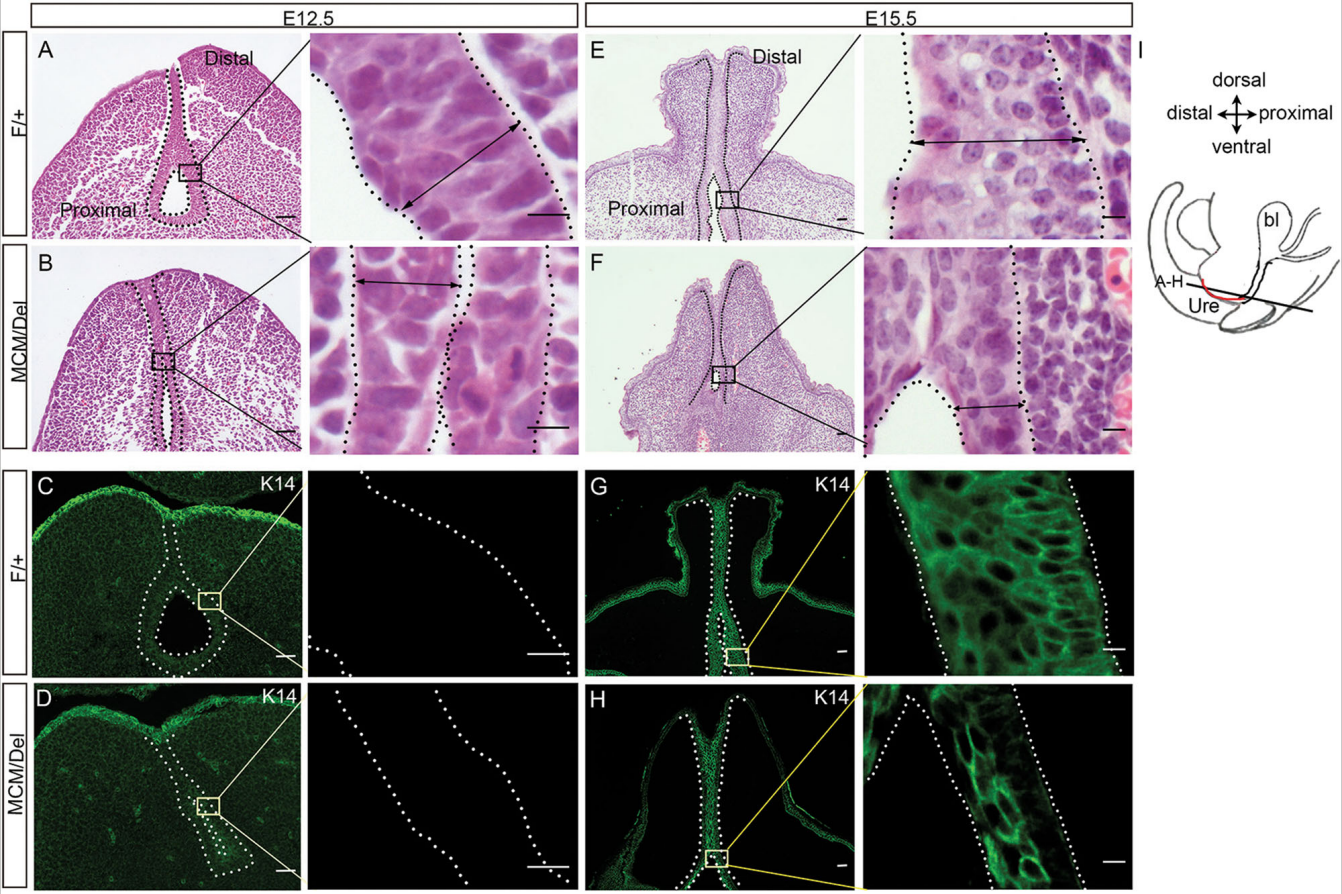
Disorganization of the urethral epithelium in male *Isl1*^*MCM/Del*^ embryos. **a**, **b**, **e**, **f** HE-stained GTs coronal sections at E12.5 and E15.5. Black double-headed arrows indicate the width of the urethral epithelium. **c**, **d**, **g**, **h** K14 expression at E12.5 and E15.5. The black or white dotted lines outlined the urethra morphology. Right are the magnified views of the frames on the left. **i** Schematic diagram of tissue slice position. Red line represents the urethral plate. Scale bars: 50 μm in **a**–**h**, 10 μm in magnified view

Formation of the GT epithelium initiates as early as E12.5^49^. In male embryos, histological examination showed that, in control embryos, basal cells were columnar and elongated perpendicular to the basement membrane, intermediate cells were smaller, and apical cells were rounded or squamous. In contrast, urethral cells in male *Isl1*^*MCM/Del*^ embryos were more homogenous, all cells being rounded, with no obvious orientation within the epithelium (Fig. 4a, b). Keratin 14 (K14), a differentiation marker, was not detected either in male controls or mutants (Fig. 4c, d). By E15.5, the GT epithelium in male controls was stratified in 4–6 cell layers, whereas in *Isl1*^*MCM/Del*^ embryos, GT epithelium was significantly thinner, being composed of 2–4 cell layers (Fig. 4e, f). In addition, in controls, K14 was evenly expressed in all urethral epithelial cells, but in *Isl1*^*MCM/Del*^ embryos, K14 was expressed only in urethral epithelium close to the lumen (Fig. 4g, h). Female embryos exhibited mutant phenotypes similar to those observed in males (data not shown). These results indicated that *Isl1* affects epithelial stratification and urethral cell differentiation.

### *Isl1* knockout enhances apoptosis of urethral epithelial cells

**Fig. 5:**
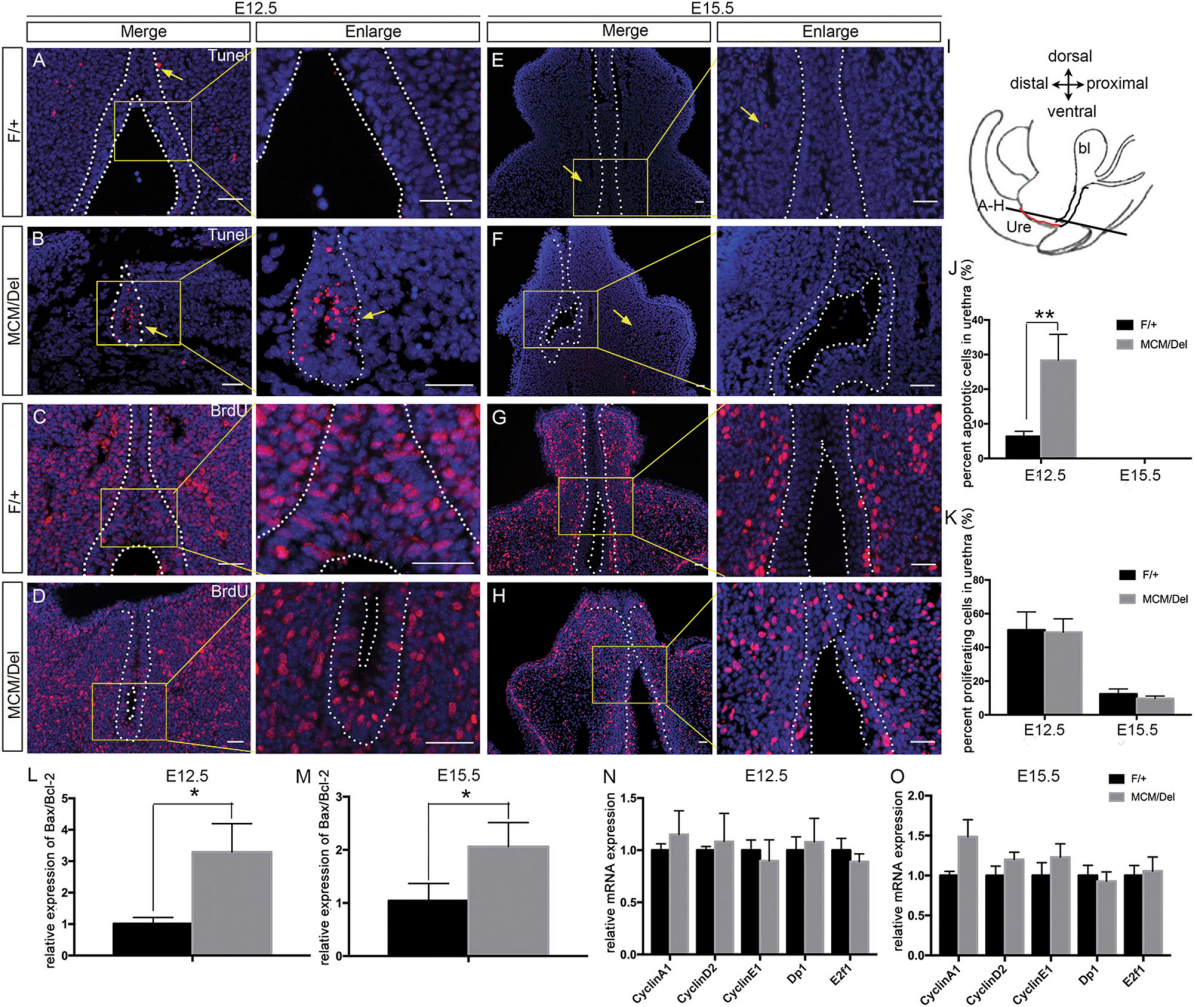
Increased cell apoptosis in urethral epithelium of *Isl1*^*MCM/Del*^ male embryos. **a**, **b**, **e**, **f** TUNEL analysis of *Isl1*^*MCM/Del*^ and control embryos at E12.5 and E15.5. Yellow arrows indicate apoptotic cells. **c**, **d**, **g**, **h** Immunofluorescence analysis of BrdU at E12.5 and E15.5 sections of *Isl1*^*MCM/Del*^ and control embryos. Right are the magnified views of the yellow frames on the left. **i** Schematic diagram of tissue slice position. Red line represents the urethral plate. **j**, **k** Percentages of apoptotic cells or BrdU-positive cells accounting for the urethral epithelial cells, respectively. **l**, **m** *Bax/Bcl-2* ratio increased in *Isl1*^*MCM/Del*^ GTs. **n**, **o** mRNA levels of cell cycle-related genes were measured by qPCR. Results were presented as means ± SEM. Each test contained at least four independent replications. *P < 0.05 and **P < 0.01. Scale bars: 50 μm

To investigate the causes by which *Isl1* ablation resulted in reductions in urethral epithelial layers, we examined urethral epithelial cell apoptosis. Results showed that the proportion of TUNEL-positive cells within epithelial cells drastically increased in *Isl1*^*MCM/Del*^ male embryos (28.3%) compared with those in control embryos (6.3%) at E12.5 (Fig. 5a, b, j). However, no TUNEL-positive cells were detected in urethral epithelium of controls or *Isl1*^*MCM/Del*^ mutants at E15.5 (Fig. 5e, f, j). In addition, we analyzed mRNA levels of the anti-apoptosis gene *Bcl-2* and the pro-apoptosis gene *Bax* by qPCR analysis of RNA isolated from whole GT tissue. Results showed that *Bax/Bcl-2* ratios rose significantly both in E12.5 and E15.5 *Isl1*^*MCM/Del*^ male embryos GTs relative to controls (Fig. 5l, m). Increased apoptosis indicated by the qPCR results at E15.5 were likely owing to inclusion of urethral mesenchymal cells, as we observed increased apoptosis in mesenchymal cells in mutants relative to controls at this stage (Fig. 5e, f).

To monitor cell proliferation, we performed BrdU labeling and immunostaining studies. In E12.5 male embryos, the proportion of BrdU-positive cells within total urethral epithelium was not significantly different between controls and mutants (49.1% versus 50.3%, Fig. 5c, d, k). The overall proportion of BrdU-positive cells significantly decreased in both controls and mutants in going from E12.5 to E15.5 (11.3% and 15.7%, respectively, Fig. 5g, h, k), consistent with a decreased proliferation rate at the later stage^10^.

Furthermore, we analyzed effects of *Isl1* expression on cell cycle genes, including *Cyclin A1*, *Cyclin D2*, *Cyclin E1*, *Dp1*, and *E2f1* in GTs of the *Isl1*^*MCM/Del*^ and control male embryos at E12.5 and E15.5. Results showed mRNA levels of these genes exhibited no significant differences between *Isl1*^*MCM/Del*^ and control male embryos (Fig. 5n, o). Similar results were observed for female embryos (data not shown).

These results indicated that *Isl1* ablation enhanced apoptosis of urethral epithelial cells at E12.5, but did not have significant effects on cell proliferation.

### ISL1 maintains urethral epithelium by targeting *Shh*

**Fig. 6:**
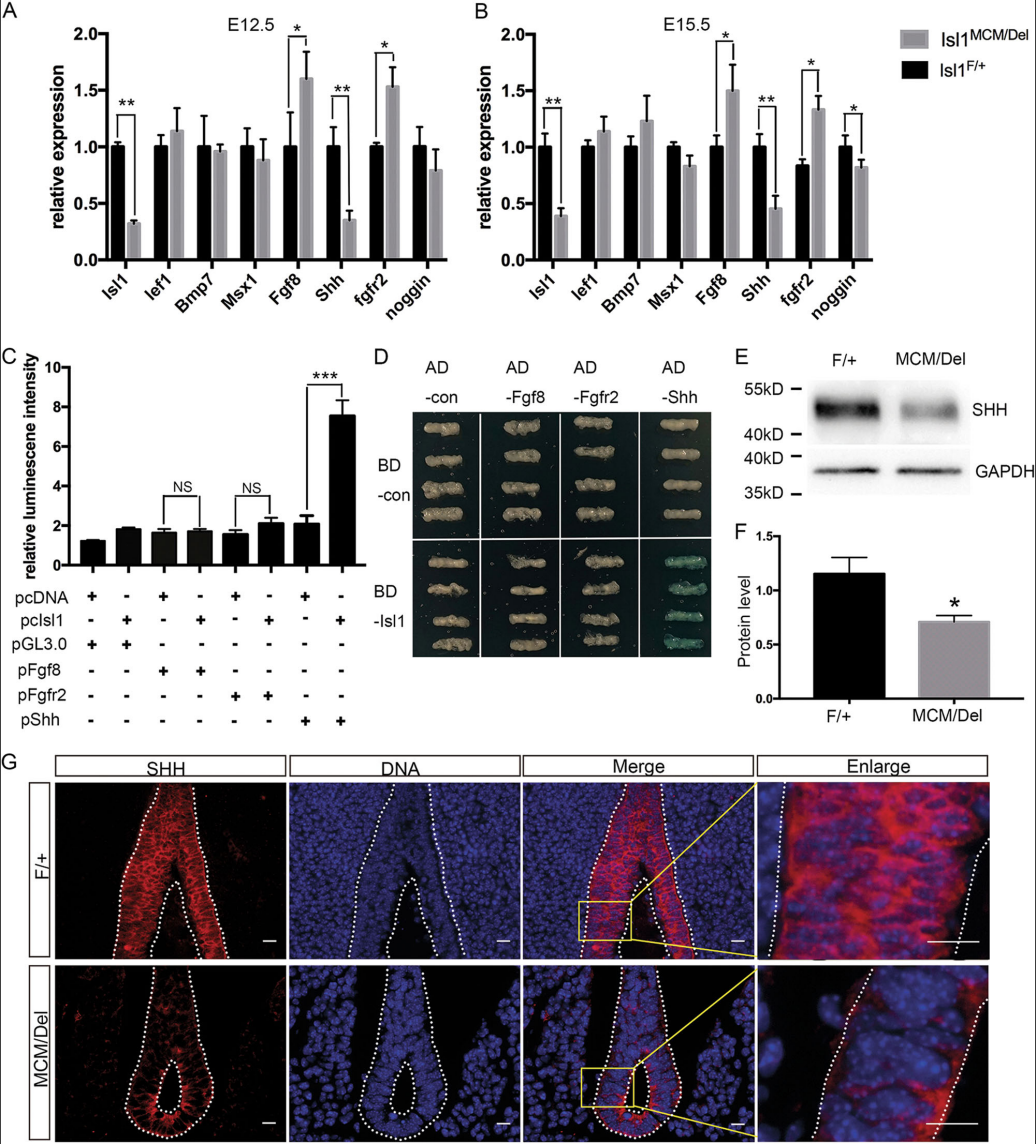
ISL1 binds to the *Shh* promoter. **a**, **b** Expression of urethral epithelium candidate genes were compared by qPCR in normal and knockout mice at E12.5 and E15.5 in male GTs. **c** Dual-Luciferase reporter assays. Only the *Shh* promoter exhibited a significant increase in relative luciferase activity in response to ISL1. **d** Yeast one-hybrid assays. BD-ISL1 activated the LacZ reporter gene driven by the *Shh* promoter fragments (AD-Shh), but not *Fgf8* (AD- Fgf8) and *Fgfr2* (AD- Fgfr2) promoter fragments. **e**, **f** SHH protein levels were examined by Western blot. **g** SHH expressed in the urethral epithelium at E12.5. The white dotted line marks the boundary of urethral epithelium. Red staining is SHH, and DAPI nuclear counterstaining (DNA) is blue. Scale bars: 20 μm. Results were presented as means ± SEM. At least four independent experiments for qPCR and Dual-Luciferase reporter assays were used. *P < 0.05, **P < 0.01, and ***P < 0.001

To find potential direct downstream targets of ISL1 affecting differentiation and apoptosis of urethral epithelial cells in mutants, we examined expression of genes required for development of urethral epithelium, including *Lef1*, *Bmp7*, *Msx1*, *Fgf8*, *Shh*, *Fgfr2*, and *Noggin*, by qPCR analyses of mRNA from mutants and controls. Results showed that levels of *Shh* mRNA were decreased in GTs of *Isl1*^*MCM/Del*^ embryos relative to controls at E12.5 and E15.5, whereas *Fgfr2* and *Fgf8* mRNA levels were significantly increased. *Isl1* ablation did not have significant effects on mRNA levels of *Lef1*, *Bmp7*, *Msx1* either at E12.5 or E15.5. At E15.5, *Noggin* mRNA levels were significantly lower in *Isl1*^*MCM/Del*^ embryos when compared with controls (Fig. 6a, b). These data suggested that *Shh*, *Fgf8*, and *Fgfr2* were potential direct downstream targets of ISL1.

To investigate whether *Shh*, *Fgf8*, and/or *Fgfr2* were direct target genes of ISL1 regulating urethral epithelium development, we performed Dual-Luciferase reporter and yeast one-hybrid assays. Promoter regions of *Shh*, *Fgf8*, and *Fgfr2* were cloned, selecting 1–2000 bp upstream of the 5′-untranslated regions as core promoter regions. Bioinformatics analyses revealed potential target sites for ISL1 (ATTA/TAAT) in promoters of all three genes. Results of Dual-Luciferase reporter assays demonstrated that ISL1 overexpression enhanced activity of the *Shh* promoter-Luciferase reporter ~3-fold, while *Fgf8* and *Fgfr2* promoter-Luciferase reporter activities exhibited no significant differences (Fig. 6c). Similarly, yeast one-hybrid assays showed that the ISL1 only activated the *Shh* promoter (Fig. 6d). In addition, Western blot results showed that SHH protein levels decreased by 40% in *Isl1*^*MCM/Del*^ embryos (Fig. 6e, f) relative to controls. Additionally, immunofluorescence analyses showed that SHH was expressed in urethral epithelial cells at E12.5, and ISL1 and SHH proteins were co-expressed within cells of the urethral epithelium. SHH protein decreased after Isl1 depletion, which was consistent with qPCR and Western blot data (Fig. 6g). Together, these data supported *Shh* as a direct target of ISL1, indicating that *Isl1* affects urethral epithelium development via *Shh*.

## Discussion

Expression and function of the LIM homeobox protein ISL1 has been extensively studied in the nervous system^23,26,50^, digestive system^24,25^, and endocrine glands^39,40^. Results of the present study demonstrated that *Isl1* was expressed in urethral epithelial cells throughout urethral development, and that ablation of *Isl1* just prior to GT development in *Isl1*^*MCM/Del*^ mutants led to GT morphological abnormalities and urethral hypoplasia.

Results indicated that *Isl1* was highly expressed in urethral epithelial cells and affected urethral formation throughout GT ontogeny. However, there is a report that conditional knockout mutants of *Isl1* using Tbx4-Cre showed GT morphological abnormalities, but no significant effect on urethral development^41^. Tbx4-Cre is expressed in mesenchymal cells beginning in early embryogenesis^51^; thus, *Isl1* was specifically knocked out in mesenchymal cells. In the study presented here, we used *Isl1-MERCreMER*^45^ induced with tamoxifen at E9.5 as the development of the urinary tract occurs after E10.5^1^ and *Isl1*-null embryos die between E7.5 and E9.5^45^. In our model, *Isl1* was knocked out both in mesenchymal and urethral epithelial cells from E9.5. Our results showed that *Isl1* deficiency resulted in GT morphological abnormalities and complete absence of the male urethra at E18.5. In support, our results are in agreement with reports that *Isl1* is expressed in mesenchymal cells derived from mesoderm, which subsequently forms the prepuce^41,42^.Thus, ISL1 is crucial for several aspects of urethral formation, as in the nervous system^23,24,50^, digestive system^25,26^, and endocrine glands systems^39,40^.

In addition, results of this study revealed that ISL1 affects urethra formation by influencing cell survival and differentiation of the epithelial cells. Histological examination revealed reduced layers of stratified urethral epithelial cells in mutants at E12.5 and E15.5, with cells closer to the lumen being more elongated in *Isl1*-knockout mice. In addition, the proportion of TUNEL-positive cells within epithelial cells was over four times higher in *Isl1*^*MCM/Del*^ male embryos than in the control in the early stage of urethra formation. However, Ching et al.^41^ reported that conditional knockout of *Isl1* in GT mesenchymal cells resulted in reduced apoptosis and had no significant effects on cell proliferation in GT^51^.

*Shh* plays important roles in regulating formation and development of multiple organs^52–57^, including urethral development^2,58^. *Shh* is specifically expressed in urethral epithelial cells, and *Shh* knockout leads to abnormal cell cycle^59^ of the urinary tract, increased cell death, and results in urethral hypoplasia^2,58^, similar to phenotypes observed with the *Isl1*^*MCM/Del*^ mice model presented here. These observations suggest that *Isl1* may affect GT development by regulating expression of *Shh*. In support, our results showed that both *Isl1* and *Shh* were expressed in all urethral epithelial cells. In addition, results of Dual-Luciferase reporter and yeast one-hybrid assays demonstrated that the *Shh* promoter contained consensus binding sites for ISL1, and that ISL1 overexpression enhanced activity of the *Shh* promoter. These results are consistent with ISL1 influencing urethral epithelium development by regulation of *Shh* expression.

Urethral hypoplasia is a common birth defect in humans, yet its etiology and pattern of onset are largely unknown. There are many factors that cause urethral hypoplasia, including environment, diet, and heredity factors. Several studies have detected significant changes in *Isl1* in human urethral hypoplasia samples compared to the normal sample by sequencing^42,60^. Draaken et al.^42^ reported that *Isl1* DNA was significantly decreased in BEEC patients, suggesting that *Isl1* plays a role in BEEC. Our laboratory studies showed that *Isl1* affected stomach pyloric sphincter development. We investigated the role of *Isl1* in the urethral sphincter, which may lead to BEEC. In addition, we tested the urethral sphincter and bladder of *Isl1*^*MCM/Del*^ mutants, and no significant abnormalities were found until E18.5 (data not shown). It may be that *Isl1*^*MCM/Del*^ mutants are not suitable for BEEC studies, but *Isl1*^*MCM/Del*^ mutants can be used as an animal model to study diseases related to urethral hypoplasia.

**Fig. 7:**
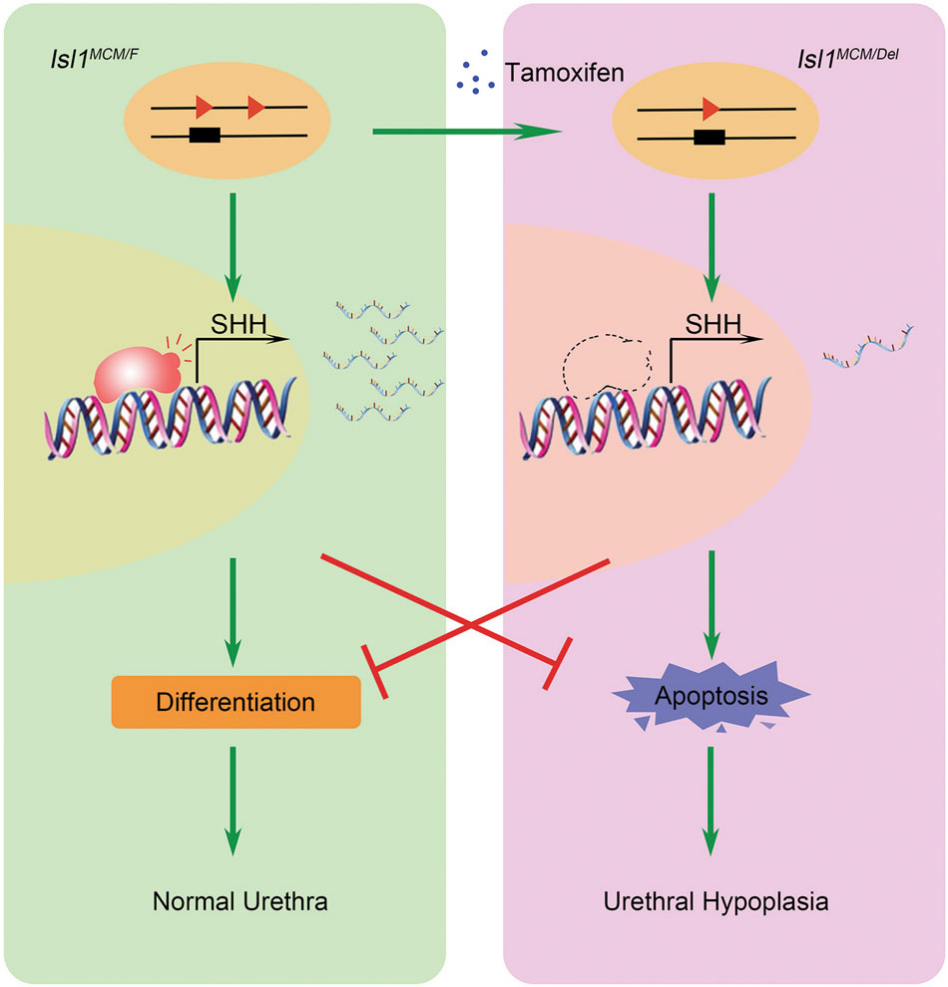
Model of *Isl1* function in mice developing urethral epithelium. Tamoxifen-induced *Isl1* DNA recombine and decreased ISL1 protein level. Insufficient ISL1 protein bind to the *Shh* gene promoter lead to failure of *Shh* transcription activation. This affects commitment of urethral epithelial cell and increasing apoptosis then resulting in hypoplasia. The green line indicates positive regulation and the red line indicates negative regulation

Lastly, we presented the novel mechanism of *Isl1* regulating urethral development (Fig. 7). In brief, *Isl1* affects urethral epithelium fate by directly targeting *Shh* promoter, subsequently affects commitment of progenitor cell, and enhance cell death. These findings are important for our understanding of diseases resulting from abnormalities of urethral development.

## Supplementary information

supplemental material

## References

[1] Haraguchi R (2000). Molecular analysis of external genitalia formation: the role of fibroblast growth factor (Fgf) genes during genital tubercle formation. Development.

[2] Lin C (2009). Temporal and spatial dissection of Shh signaling in genital tubercle development. Development.

[3] Qian Chen, Wu Zhongluan, Ng Roy Chun-Laam, Garcia-Barceló Maria-Mercè, Yuan Zheng-Wei, Wong Kenneth Kak Yuen, Tam Paul Kwong Hang, Lui Vincent Chi Hang (2018). Conditional deletion of platelet derived growth factor receptor alpha (Pdgfra) in urorectal mesenchyme causes mesenchyme apoptosis and urorectal developmental anomalies in mice. Cell Death & Differentiation.

[4] Matsushita S (2018). Regulation of masculinization: androgen signalling for external genitalia development. Nat. Rev. Urol..

[5] Baskin L (2001). Urethral seam formation and hypospadias. Cell Tissue Res..

[6] Suzuki K (2003). Regulation of outgrowth and apoptosis for the terminal appendage: external genitalia development by concerted actions of BMP signaling [corrected]. Development.

[7] Perriton CL, Powles N, Chiang C, Maconochie MK, Cohn MJ (2002). Sonic hedgehog signaling from the urethral epithelium controls external genital development. Dev. Biol..

[8] Cohn MJ (2011). Development of the external genitalia: conserved and divergent mechanisms of appendage patterning. Dev. Dyn..

[9] Herrera AM, Cohn MJ (2014). Embryonic origin and compartmental organization of the external genitalia. Sci. Rep..

[10] Petiot A, Perriton CL, Dickson C, Cohn MJ (2005). Development of the mammalian urethra is controlled by Fgfr2-IIIb. Development.

[11] Cunha GR, Sinclair A, Risbridger G, Hutson J, Baskin LS (2015). Current understanding of hypospadias: relevance of animal models. Nat. Rev. Urol..

[12] Shiroyanagi Y (2007). Urothelial sonic hedgehog signaling plays an important role in bladder smooth muscle formation. Differentiation.

[13] Cheng W (2008). Sonic hedgehog mediator Gli2 regulates bladder mesenchymal patterning. J. Urol..

[14] Haraguchi R (2012). The hedgehog signal induced modulation of bone morphogenetic protein signaling: an essential signaling relay for urinary tract morphogenesis. PLoS ONE.

[15] Ng RC (2014). Dysregulation of Wnt inhibitory factor 1 (Wif1) expression resulted in aberrant Wnt-b-catenin signaling and cell death of the cloaca endoderm, and anorectal malformations. Cell Death Differ..

[16] Morgan EA (2003). Loss of Bmp7 and Fgf8 signaling in Hoxa13-mutant mice causes hypospadia. Development.

[17] Seifert AW, Yamaguchi T, Cohn MJ (2009). Functional and phylogenetic analysis shows that Fgf8 is a marker of genital induction in mammals but is not required for external genital development. Development.

[18] Gredler ML, Seifert AW, Cohn MJ (2015). Tissue-specific roles of Fgfr2 in development of the external genitalia. Development.

[19] Ikeda Y (2017). Fgfr2 is integral for bladder mesenchyme patterning and function. Am. J. Physiol. Ren. Physiol..

[20] Lin C, Yin Y, Long F, Ma L (2008). Tissue-specific requirements of beta-catenin in external genitalia development. Development.

[21] Lin C (2013). Delineating a conserved genetic cassette promoting outgrowth of body appendages. PLoS Genet..

[22] Miyagawa S (2014). Disruption of the temporally regulated cloaca endodermal beta-catenin signaling causes anorectal malformations. Cell Death Differ..

[23] Kim KT (2016). ISL1-based LIM complexes control Slit2 transcription in developing cranial motor neurons. Sci. Rep..

[24] Zhang Q (2018). Temporal requirements for ISL1 in sympathetic neuron proliferation, differentiation, and diversification. Cell Death Dis..

[25] Li Y (2014). LIM homeodomain transcription factor Isl1 directs normal pyloric development by targeting Gata3. BMC Biol..

[26] Guo T (2019). ISL1 predicts poor outcomes for patients with gastric cancer and drives tumor progression through binding to the ZEB1 promoter together with SETD7. Cell Death Dis..

[27] Suzuki K (2012). Reduced BMP signaling results in hindlimb fusion with lethal pelvic/urogenital organ aplasia: a new mouse model of sirenomelia. PLoS ONE.

[28] Tahara N (2018). Gata6 restricts Isl1 to the posterior of nascent hindlimb buds through Isl1 *cis*-regulatory modules. Dev. Biol..

[29] Yang L (2006). Isl1Cre reveals a common Bmp pathway in heart and limb development. Development.

[30] Gao R (2019). Pioneering function of Isl1 in the epigenetic control of cardiomyocyte cell fate. Cell Res..

[31] Witzel HR (2012). The LIM protein Ajuba restricts the second heart field progenitor pool by regulating Isl1 activity. Dev. Cell.

[32] Lin L (2007). Beta-catenin directly regulates Islet1 expression in cardiovascular progenitors and is required for multiple aspects of cardiogenesis. Proc. Natl Acad. Sci. USA.

[33] Laugwitz KL, Moretti A, Caron L, Nakano A, Chien KR (2008). Islet1 cardiovascular progenitors: a single source for heart lineages?. Development.

[34] Elshatory Y, Deng M, Xie X, Gan L (2007). Expression of the LIM-homeodomain protein Isl1 in the developing and mature mouse retina. J. Comp. Neurol..

[35] Bejarano-Escobar R (2015). Expression and function of the LIM-homeodomain transcription factor islet-1 in the developing and mature vertebrate retina. Exp. Eye Res..

[36] Ediger BN (2014). Islet-1 Is essential for pancreatic beta-cell function. Diabetes.

[37] Yan C (2016). Protein Inhibitor of activated STAT Y (PIASy) regulates insulin secretion by interacting with LIM homeodomain transcription factor Isl1. Sci. Rep..

[38] Wu Y (2010). LIM homeodomain transcription factor Isl-1 enhances follicle stimulating hormone-beta and luteinizing hormone-beta gene expression and mediates the activation of leptin on gonadotropin synthesis. Endocrinology.

[39] Zhang J (2018). LIM homeobox transcription factor Isl1 is required for melatonin synthesis in the pig pineal gland. J. Pineal Res..

[40] Qiu J (2019). MicroRNA-7 inhibits melatonin synthesis by acting as a linking molecule between leptin and norepinephrine signaling pathways in pig pineal gland. J. Pineal Res..

[41] Ching ST (2018). Isl1 mediates mesenchymal expansion in the developing external genitalia via regulation of Bmp4, Fgf10 and Wnt5a. Hum. Mol. Genet.

[42] Draaken M (2015). Genome-wide association study and meta-analysis identify ISL1 as genome-wide significant susceptibility gene for bladder exstrophy. PLoS Genet..

[43] Zhang R (2017). ISL1 is a major susceptibility gene for classic bladder exstrophy and a regulator of urinary tract development. Sci. Rep..

[44] Kaku Y (2013). Islet1 deletion causes kidney agenesis and hydroureter resembling CAKUT. J. Am. Soc. Nephrol..

[45] Laugwitz KL (2005). Postnatal isl1+ cardioblasts enter fully differentiated cardiomyocyte lineages. Nature.

[46] Tunster SJ (2017). Genetic sex determination of mice by simplex PCR. Biol. Sex Differ..

[47] Alasaad S (2008). HotSHOT Plus ThermalSHOCK, a new and efficient technique for preparation of PCR-quality mite genomic DNA. Parasitol. Res..

[48] Sun Y (2008). A central role for Islet1 in sensory neuron development linking sensory and spinal gene regulatory programs. Nat. Neurosci..

[49] Georgas KM (2015). An illustrated anatomical ontology of the developing mouse lower urogenital tract. Development.

[50] Huber K (2013). The LIM-Homeodomain transcription factor Islet-1 is required for the development of sympathetic neurons and adrenal chromaffin cells. Dev. Biol..

[51] Luria V, Krawchuk D, Jessell TM, Laufer E, Kania A (2008). Specification of motor axon trajectory by ephrin-B:EphB signaling: symmetrical control of axonal patterning in the developing limb. Neuron.

[52] Zhang L (2013). Sonic hedgehog signaling pathway mediates cerebrolysin-improved neurological function after stroke. Stroke.

[53] Qin S (2019). Downregulation of sonic hedgehog signaling in the hippocampus leads to neuronal apoptosis in high-fat diet-fed mice. Behav. Brain Res..

[54] Wang Y, Peng Q, Jia H, Du X (2016). Prognostic value of hedgehog signaling pathway in digestive system cancers: a systematic review and meta-analysis. Cancer Biomark..

[55] Konstantinou D, Bertaux-Skeirik N, Zavros Y (2016). Hedgehog signaling in the stomach. Curr. Opin. Pharmacol..

[56] Jimenez-Caliani AJ (2017). αE-catenin is a positive regulator of pancreatic islet cell lineage. Differ. Cell Rep..

[57] Mfopou JK, De Groote V, Xu X, Heimberg H, Bouwens L (2007). Sonic hedgehog and other soluble factors from differentiating embryoid bodies inhibit pancreas development. Stem Cells.

[58] Miyagawa S (2009). Dosage-dependent hedgehog signals integrated with Wnt/beta-catenin signaling regulate external genitalia formation as an appendicular program. Development.

[59] Seifert AW, Zheng Z, Ormerod BK, Cohn MJ (2010). Sonic hedgehog controls growth of external genitalia by regulating cell cycle kinetics. Nat. Commun..

[60] Arkani S (2018). Evaluation of the ISL1 gene in the pathogenesis of bladder exstrophy in a Swedish cohort. Hum. Genome Var..

