# Supplementary material for: LIM homeodomain transcription factor Isl1 affects urethral epithelium differentiation and apoptosis via Shh
Source: Cell Death Dis. 2019 Sep 26;10(10):713. doi: 10.1038/s41419-019-1952-z (PMC6763423; doi:10.1038/s41419-019-1952-z)
Supplement: Supplementary file 1 — supplemental material [file 41419_2019_1952_MOESM1_ESM.doc]

**Supplementary Table S1.** qPCR primer sequences

| **Gene** | **Forward primers** | **Reverse primers** |
| --- | --- | --- |
| ***Gapdh*** | GGTTGTCTCCTG CGACTTCA | GGGTGGTCCAGGGTTTCTTA |
| ***Isl1*** | CTGCACACCTTGCGGACCT | ACACTCGGATGACTCTGGGAC |
| ***Shh*** | GAGGGAACGAACGAGCCG | ACTTGTCTTTGCACCTCTGAGTC |
| ***Fgfr2*** | GAGTCCAGCTCCTCCATGAAC | GCGTCAGCTTATCTCTGGGG |
| ***Fgf8*** | CGAGCACGACATTCCACGAG | CAAGTGCAACAGCAGGCAG |

Primers sequences for identifying gene expression change in *Isl1^MCM/Del^* mouse embryos.

**Supplementary Table S2.** Sequences of primers used for genotype identification

| **Gene** | **Sequences** |
| --- | --- |
| ***MCM-F*** | ACTATTTGCCACCTAGCCACAGCA |
| ***MCM-R*** | GTTCAGCATCCAACAAGGCACTGA |
| ***F1*** | GGTCTCTGGAACATCCCACAT |
| ***R1*** | CTGTTCCTACTCCCCATTCACT |
| ***R2*** | CCAGTCTCGGTCTGCCTGCC |

**Supplementary Table S3**. Sequences of primers used for Dual-Luciferase reporter assays

| **Gene** | **Forward primers** | **Reverse primers** |
| --- | --- | --- |
| ***Isl1-DL*** | CGGCTAGCCAGATA  TGGGAGACATGGG | ATAGGACTGGCTACC  ATGCTGCTCGAGGG |
| ***Shh-DL*** | CGACGCGTCTCGAG  ACCCAACTCCGATG | CCCAAGCTTCGCAGA  GTTAACTGTAGCTCG |
| ***Fgfr2-DL*** | CTAGCTAGCCGCTG  CCTCTGACGTTAGTT | CCGCTCGAGGGAAGG  GAACACGTGAATAGGT |
| ***Fgf8-DL*** | CTAGCTAGCGACAGA  CTGCTTCCTGATCCC | CCGCTCGAGGGAGGC  CAGCGGCTATC |

Primers sequences for constructing plasmid for Luciferase assays.

**Supplementary Table S4**. Sequences of primers used for Yeast one-hybrid assay

| **Gene** | **Forward primers** | **Reverse primers** |
| --- | --- | --- |
| ***BD-Isl1*** | GATTATGCCTCTCCCAT  ATGGGAGACATGGGCGAT | CGAAGAAGTCCAAAGCTTT  CATGCCTCAATAGGACTGG |
| ***AD-Shh*** | TCGGAATTCGAGCTCTC  TCCAGCCTTGCTACCATTT | AGCACATGCCTCGAGCGC  GGTCCAGATTTAAGGTG |
| ***AD-Fgfr2*** | TCGGAATTCGAGCTCCG  CTGCCTCTGACGTTAGTT | AGCACATGCCTCGAGGGA  AGGGAACACGTGAATAGGT |
| ***AD-Fgf8*** | TCGGAATTCGAGCTCGAC  AGACTGCTTCCTGATCCC | AGCACATGCCTCGAGGGA  GGCCAGCGGCTATC |

Primers sequences for constructing plasmid for Yeast one-hybrid assay.
